# Accurate Energy Modeling and Characterization of IEEE 802.11ah RAW and TWT

**DOI:** 10.3390/s19112614

**Published:** 2019-06-08

**Authors:** Serena Santi, Le Tian, Evgeny Khorov, Jeroen Famaey

**Affiliations:** 1 Internet and Data Lab (IDLab), Department of Mathematics and Computer Science, University of Antwerp—imec, 2000 Antwerp, Belgium; le.tian@uantwerpen.be (L.T.); jeroen.famaey@uantwerpen.be (J.F.); 2 Wireless Networks Lab, Institute for Information Transmission Problems, Russian Academy of Sciences, 127051 Moscow, Russia; e@khorov.ru

**Keywords:** internet of things, IEEE 802.11ah, Wi-Fi HaLow, energy consumption, RAW, TWT

## Abstract

Minimizing the energy consumption is one of the main challenges in internet of things (IoT) networks. Recently, the IEEE 802.11ah standard has been released as a new low-power Wi-Fi solution. It has several features, such as restricted access window (RAW) and target wake time (TWT), that promise to improve energy consumption. Specifically, in this article we study how to reduce the energy consumption thanks to RAW and TWT. In order to do this, we first present an analytical model that calculates the average energy consumption during a RAW slot. We compare these results to the IEEE 802.11ah simulator that we have extended for this scope with an energy life-cycle model for RAW and TWT. Then we study the energy consumption under different conditions using RAW. Finally, we evaluate the energy consumption using TWT. In the results, we show that the presented model has a maximum deviation from the simulations of 10% in case of capture effect (CE) and 7% without it. RAW always performs better than carrier-sense multiple access with collision avoidance (CSMA/CA) when the traffic is higher and the usage of more slots has showed to have better energy efficiency, of up to the 76%, although also significantly increasing the latency. We will show how TWT outperforms pure RAW, by over 100%, when the transmission time is over 5 min.

## 1. Introduction

Many companies predict that the internet of things (IoT) will consist of dozens of billions of devices connected together over the Internet. Currently, IoT offers a large number of solutions to connect devices with each other [1]. The recently released IEEE 802.11ah standard, marketed as Wi-Fi HaLow, allows the connection of up to 8192 devices with one access point (AP), combining the advantages of Wi-Fi and low-power communication technologies and operating in the unlicensed sub-1-GHz frequency bands (e.g., 863–868 MHz in Europe and 902–928 MHz in North America). Its main characteristic is to provide a good trade-off between range, throughput, and energy efficiency. On the Media Access Control Layer (MAC), it offers different mechanisms to support power-limited stations (STAs) in dense networks, such as hierarchical organization, short MAC header, fast association, restricted access window (RAW), traffic indication map (TIM) and target wake time (TWT).

Although the standard is promising, the hardware is not on the mass market yet. However, researchers have been investigating the standard for a few years already. Early research was based on analytical modeling of the saturated network state, which does not accurately capture realistic IoT network behavior and is arduous to adapt to non-saturated network conditions.

To evaluate IEEE 802.11ah more realistically, we implemented the standard in the ns-3 network simulator [2,3]. This simulator has many features introduced by the IEEE 802.11ah standard, such as sub-1GHz channel models, adaptive modulation and coding scheme (MCS), fast association, RAW, TIM segmentation and TWT. Particularly, RAW aims to reduce collisions in dense IoT networks, where thousands of STAs are connected to one AP, by dividing them into groups and only allowing one group to access the channel in specific intervals, or during shared slots that all the STAs can access. Moreover, TWT helps to reduce the energy consumption by letting STAs sleep and only waking up to transmit their packets, skipping multiple beacons without being disassociated from the network.

Existing research has focused on various metrics for the evaluation of this standard, such as throughput and latency, using analytical models and other tools for the validation. However, this standard is intended to be used in IoT networks where the energy consumption is of paramount importance.

To address this, we present four contributions. First, we present an extension to the analytical model of Khorov et al. [4]. This model calculates the required time needed for the STAs to send their frames, which can be used for an estimation of the appropriate RAW slot duration. We extend this model in order to calculate the energy consumption given a fixed slot duration. Second, we implement an energy life-cycle model in the ns-3 simulator in order to allow STAs to enter a sleep state and to store the timing information of all states (Receive, Transmit, Idle, Collision, Sleep), that are used to calculate the energy consumption. We compare the results of the extension of the analytical model and of the ns-3 simulator in order to validate our model. Third, we compare various RAW configurations to study the effect of various parameters on energy consumption. Fourth, we extend the ns-3 simulator adding the TWT feature, and evaluate its energy consumption compared to the RAW mechanism.

The rest of the article is organized as follows. Section 2 introduces related research on IEEE 802.11ah energy characterization. Section 3 provides an overview of the implementation of the RAW energy model in the ns-3 simulator. The description of the extensions of the analytical model is presented in Section 4. In Section 5, we provide a comparison of the results of the model together with the results of the simulations, and also evaluate RAW and TWT in terms of energy efficiency. Finally, conclusions and future work are discussed in Section 6.

## 2. Related Work

The IEEE 802.11ah standard made its first appearance in October 2013 and was officially released in June 2017. Since then, researchers have been investigating the advantages and challenges in the design of Physical Layer (PHY) and MAC layer schemes [5,6,7,8,9,10]. To date, the research community has been interested in evaluating some of the key features of this technology, such as RAW.

However, the evaluation has been done mostly using analytical models [4,11,12,13,14]. More relevant to the research presented in this article is the work focusing on the energy consumption of RAW. Several studies have been done on the calculation of the energy consumption of RAW and TIM, given specific network and traffic conditions [11,15,16,17,18]. Raeesi et al. present an analytical model to compute the energy consumption and the throughput of IEEE 802.11ah [11], however, they only consider saturated traffic scenarios. Park evaluates an initial version of RAW using synch frames, providing results about energy consumption and delay, without considering collisions [16]. Zheng et al. present an analytical model to calculate the throughput for RAW in a saturated network, performing various analysis with different group configurations, considering both cross and non-cross slot boundaries [18]. Khorov et al. have recently presented a new mathematical model to allow the estimation of throughput and energy consumption using RAW with cross slot boundary (CSB) enabled [19], however, they consider a set of STAs transmitting saturated uplink data. Beltramelli et al. evaluate the delay and the energy consumption using a hybrid MAC mechanism where the STAs first notify the AP when they have a buffered packet to send, during a contention phase [12]. In our model, we assume a network where the STAs have at most one packet to send every beacon interval, since this is more realistic than saturated conditions given the sporadic traffic in sensor networks.

Tian et al. propose an algorithm for real-time STA grouping, evaluating it in terms of throughput, packet loss and latency, using dynamic traffic [15]. Slijvo et al. study how different parameters can affect RAW and TIM, considering bidirectional traffic and providing the evaluation of throughput, latency and sleep time [20]. Bel et al. analyze energy consumption focusing on TIM and page segmentation, using a low traffic load scenario making this work complementary to our study [21], as we study RAW and TWT instead of TIM. Zhao et al., similarly to Bel et al. [21], evaluated the energy consumption of IEEE 802.11ah focusing on TIM using a system-level MAC simulator assuming an uplink scenario with sporadic data traffic [17].

To our knowledge, only the research done by Beltramelli et al. [12] evaluated the TWT feature, using an analytical model and comparing it to RAW. However, they do not consider the impact of sleeping time on the results.

In this paper, we present an analytical model that allows the evaluation of the energy consumption in an IEEE 802.11ah network, using RAW and TWT. We compare the results between the model and the ns-3 simulator [3], studying scenarios with different types of traffic, different configurations and capture effect (CE). Moreover, we present the first results of the implementation of TWT in our simulator, evaluating it in comparison to RAW. Our results are more accurate because we consider the energy consumed during all device states as well as accurate beacon and header overhead.

## 3. Restricted Access Window and Target Wake Time Energy State Model

**Figure 1:**
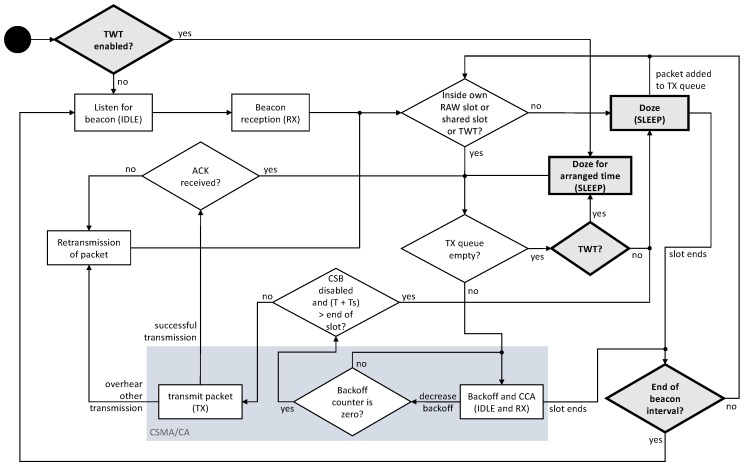
Radio state diagram for the developed energy model of IEEE 802.11ah with restricted access window (RAW) and target wake time (TWT).

This section presents RAW and TWT, two main features of the 802.11ah standard that allow better management of the energy consumption in power constrained STAs. The bold boxes with the grey background in Figure 1 show our contribution, namely the addition to the ns-3 simulator of the energy state model that allows proper sleep-cycles, the storing of the timings for each state of each STA and the TWT feature.

### 3.1. Restricted Access Window (RAW)

Numerous studies have shown that traditional channel access methods such as carrier-sense multiple access with collision avoidance (CSMA/CA) and time-division multiple access (TDMA) are not suitable for dense IoT networks, due to lack of scalability and increase of delays [22]. For this reason, a more flexible paradigm has emerged, referred to as STA grouping, and introduced by IEEE 802.11ah. It combines the advantages of CSMA/CA and TDMA.

**Figure 2:**
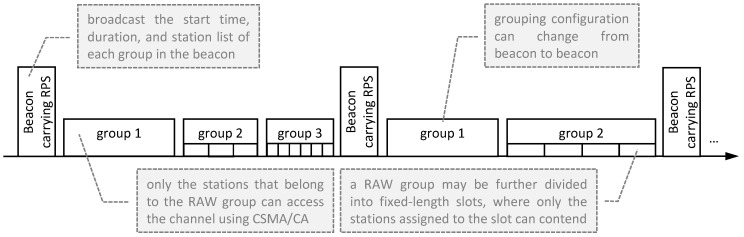
Schematic representation of the RAW mechanism.

As Figure 2 shows, STAs are split into groups, and only one group can access the channel during a specific interval using CSMA/CA. Groups can be further split into fixed-duration slots in order to reduce the number of contending STAs even more. During a RAW slot, only the STAs belonging to that slot are allowed to access the channel. So as to manage the grouping information, the AP broadcasts a beacon which contains the RAW parameter set (RPS) every fixed-length interval. The RPS contains the necessary configuration parameters, such as the number of groups, each group start time, duration, number of slots per groups and assigned STA list. The STAs belonging to a RAW group have sequential association ID (AID) and are assigned to RAW slots in a round-robin fashion.

As the standard does not suggest any algorithm to manage these parameters, there is a lot of flexibility in order to manage the grouping configuration. This has many advantages, such as an increase in energy efficiency and scalability and reduction of contention and collisions. Another advantage is introduced by the fact that the configuration can be adjusted from beacon to beacon, with the possibility to adapt the configuration to the actual network dynamics.

Figure 1 depicts all energy state transitions in IEEE 802.11ah using RAW and TWT. At the beginning of the beacon interval the STA, if it does not have TWT enabled, goes into the idle state, waiting to receive the beacon from the AP. From the beacon, the STA learns when its RAW slot and any shared slot starts and ends. After receiving the beacon, the STA goes into doze state, until it arrives to its RAW slot or into a shared slot. Then the STA checks if there are packets to be sent. In case there are, it uses CSMA/CA to transmit its data. This process has a blue-grey background in Figure 1. Also if the packet arrives while the slot has already begun, the STA checks if there is enough time to send it, in case CSB is enabled. Otherwise, the STA remains in the doze state until the next slot or until next beacon. At the end of the beacon interval, the STA goes in the doze state until it wakes up to receive the new beacon, after which the process repeats.

Based on this state diagram, we have developed the state transition model for RAW in the 802.11ah ns-3 module [3]. We developed several extensions, allowing us to measure the timings of the radio states of the STAs and to allow STAs to go into doze state outside their transmission or reception periods. These changes are available in the published module as open source (https://github.com/imec-idlab/IEEE-802.11ah-ns-3).

### 3.2. Target Wake Time (TWT)

TWT is a mechanism that allows STAs to agree with the AP on the possibility to remain into the doze state, skipping the reception of multiple beacons, without being disassociated from the AP. This mechanism is also present in the IEEE 802.11ax standard [23,24,25].

The STAs and the AP exchange information that includes the time length during which the STA will be in the doze state. This exchange allows the AP to know when the STAs are going to be awake, in order to send buffered data. TWT may be used to reduce energy consumption, as STAs that use it can enter into the doze state for an agreed interval duration, without losing association with the AP. As a result, STAs operate at different times in order to minimize contention and reduce the required amount of time a STA needs to be awake [26].

Figure 1 shows how this mechanism is implemented in the simulator. In the beginning, if TWT is enabled, the STA sleeps for the agreed time, with the possibility of skipping multiple beacons, until it wakes up in order to send its buffered packets, then the process continues as with RAW until all the packets are sent. Then, the STA goes back into the doze state.

## 4. Energy Consumption Markov Process for RAW

This section presents a Markov process that models the cumulative energy consumption of more STAs competing for the channel within a fixed duration RAW slot. The process follows the state transition rules defined in the state diagram presented in Figure 1 and it models the energy consumption of all STAs being part of the RAW slot. The presented model extends the model previously proposed by Khorov et al. [4]. In contrast to Khorov, we model energy consumption in addition to the throughput, and assume a fixed-size RAW slot, rather than calculating a transmission probability distribution as a function of RAW slot duration. Our presented model supports only non-CSB (Cross Slot Boundary). In fact, in recent work it is shown [19] how enabling the CSB affects the throughput and the power consumption. In line with Khorov’s model, a machine-type communication scenario is assumed, where each STA attempts to transmit exactly 1 frame per slot. Moreover, it is assumed there are no hidden nodes, no (CE), the AP only transmits acknowledgements (ACKs) and only two STAs can collide, to limit the complexity of the model.

Table 1 introduces the notations and symbols used throughout this section. The duration of the different radio states used in the model are depicted in Table 2; these durations are based on the IEEE 802.11ah standard [26]. In contrast to the original model, we model $T_{tx}$ as a function of the MCS data rate and payload size. As such, the proposed model is applicable to STAs with heterogeneous data rate and frame size.

### Process

This process calculates the total energy consumption of all the STAs that transmit in the RAW slot. Its state is modelled as $\left(t,c,s\right)$, with *t* the index of the current time slot, *c* the number of collisions of all the packets in the network, and *s* the number of all the successful transmissions. The index of the current time slot, *t*, is used as an abstract time in this process. The real-time duration of each abstract time slot depends on its state: collision, idle or successful, and can be found in Table 2. Based on the number of collision, idle and successful transitions, the real amount of passed time can then be calculated as follows:

$$T\left(t,c,s\right)=c\times T_{c}+s\times T_{s}+\left(t-c-s\right)\times T_{e}.$$

**Figure 3:**
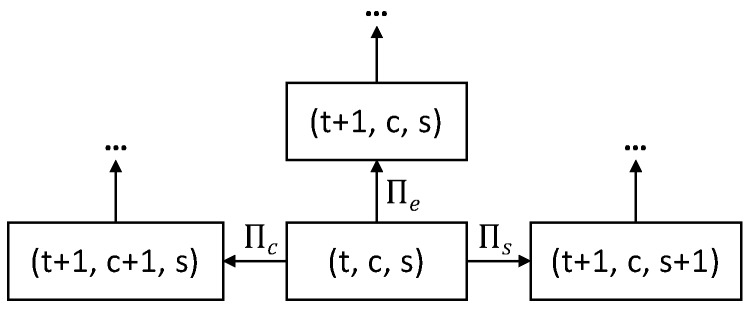
Diagram of the process.

A STA can reach three different states: (i) when it does not perform any operation (reachable with transition $\Pi_{e}$), (ii) when the STA has successfully transmitted its packet (reachable with transition $\Pi_{s}$), and (iii) when the transmission of the STA has collided with the transmission of another STA (reachable with transition $\Pi_{c}$). The process starts in state $\left(0,0,0\right)$. As Figure 3 shows, from state $\left(t,c,s\right)$ three transitions are possible:

1. With transition probability $\Pi_{e}\left(t,c,s\right)$, slot *t* is empty, the process transitions to $\left(t+1,c,s\right)$ with energy consumption:
  $$E_{e}=P_{idle}\times T_{e}\times \left(N-s\right)+P_{sleep}\times T_{e}\times s.$$
2. With transition probability $\Pi_{s}\left(t,c,s\right)$, the transmission attempt of a STA at time *t* is successful. As such, the STA does not have any other frame to transmit and goes into the sleep state. The other STAs that have packets to transmit remain in their current state and the counters are updated. If all the STAs (i.e., s+1=N) successfully transmit their packet, the process goes into the successful absorbing state. The process transitions to $\left(t+1,c,s+1\right)$, with energy consumption:
  $$\begin{array}{cc} E_{s}= & \left(P_{rx}\times \left(T_{tx}+T_{ack}\right)+P_{idle}\times \left(T_{s}-T_{tx}-T_{ack}\right)\right)\times \left(N-s-1\right)\\ & +P_{tx}\times T_{tx}+P_{rx}\times T_{ack}+P_{idle}\times \left(T_{s}-T_{tx}-T_{ack}\right)+P_{sleep}\times T_{s}\times s. \end{array}$$
3. With transition probability $\Pi_{c}\left(t,c,s\right)$, a collision occurs at time *t*, this means that two STAs try to transmit. The process transitions to $\left(t+1,c+1,s\right)$, with energy consumption
  $$\begin{array}{cc} E_{c}= & \left(P_{rx}\times T_{tx}+P_{idle}\times \left(T_{c}-T_{tx}\right)\right)\times \left(N-s-2\right)\\ & +2\times \left(P_{tx}\times T_{tx}+P_{idle}\times \left(T_{c}-T_{tx}\right)\right)+P_{sleep}\times T_{c}\times s. \end{array}$$

Moreover, any transition can result in the unsuccessful absorbing state if the end of the RAW slot has been reached. The actual stop condition, namely $t+T_{s}\geq T_{slot}$, is based on the fact that the IEEE 802.11ah CSB feature is not enabled.

Based on the energy consumption calculation for each possible transition from state $\left(t,c,s\right)$, the total energy consumption for all STAs in a slot starting from state $E_{\left(0,0,0\right)}$ onwards can be recursively calculated as follows:

(1) $$\begin{array}{cc} E_{\left(t,c,s\right)}= & \Pi_{e}\left(t,c,s\right)\times \left(E_{e}+E_{\left(t+1,c,s\right)}\right)\\ & +\Pi_{s}\left(t,c,s\right)\times \left(E_{s}+E_{\left(t+1,c,s+1\right)}\right)+\Pi_{c}\left(t,c,s\right)\times \left(E_{c}+E_{\left(t+1,c+1,s\right)}\right). \end{array}$$

For any unsuccessful absorbing state $\left(t,c,s\right)$, the energy consumption $E_{\left(t,c,s\right)}$ equals 0. The total energy consumption of all *N* contending STAs within a RAW slot of duration $T_{slot}$, can then be recursively calculated from state $E_{\left(0,0,0\right)}$.

The transition probabilities used in this model ($\Pi_{e}$, $\Pi_{s}$ and $\Pi_{c}$) are identical to those in the model previously proposed by Khorov et al. [4] and are as follows:

$$\Pi_{e}\left(t,c,s\right)={\left(1-Pr\left(TX|t,c,s\right)\right)}^{N-s},$$

$$\Pi_{s}\left(t,c,s\right)=\left(N-s\right)Pr\left(TX|t,c,s,\right)\times {\left(1-Pr\left(TX|t,c,s\right)\right)}^{N-s-1},$$

$$\Pi_{c}\left(t,c,s\right)=1-\Pi_{e}-\Pi_{s}.$$

## 5. Results and Evaluation

This section presents the results of the energy model implemented in our simulation framework [3] and of the extension to the analytical model proposed by Khorov et al. [4]. In the first part, we present the results of the comparison between our analytical model and the energy consumption model implemented in the ns-3 simulator. Then, analyze the energy consumption of RAW using the simulation model. Finally, we report the energy consumption results using the TWT feature.

### 5.1. Simulation Set-Up and Energy Model Implementation in ns-3

Using the existing IEEE 802.11ah implementation in ns-3 [3], some modifications were applied in order to measure the energy consumed and to allow devices to go into a sleep state outside their transmission or reception periods. Figure 1 depicts the potential state transitions in IEEE 802.11ah using RAW and TWT. Based on this state diagram, we implemented the state transition of the device to the sleep state in the IEEE 802.11ah ns-3 module. When CSMA/CA was used, STAs woke up to receive the beacon, then they contend for the channel. After they have transmitted all queued packets, they go in the sleep state.

Then, we calculated how much time each device spends in the transmit, receive, idle, collide and sleep state in order to calculate the energy consumption, where the colliding state represents unsuccessful TX transmissions, while the transmit state represents successful ones. As no off-the-shelf IEEE 802.11ah hardware is available, we used the energy consumption values of the AT86RF215 Atmel radio [27] in the results, because it uses similar PHY modulations and it also covers sub-1-GHz frequencies.

All evaluations were performed using the parameters in Table 3, unless specified differently, and focusing on 1 MHz channel bandwidth, as this was most relevant for IoT applications. If CE was enabled in the simulator, the AP was able to successfully detect and decode a packet while it is receiving another one, if the reception power difference between both packets is high enough. If CE is disabled, colliding packets are always discarded.

Each experiment was repeated 10 times. The error bars in the graphs depict the standard deviation across these repetitions. The Poisson packet arrival rate, location of devices, and back-off timers are randomized across repeated experiments.

### 5.2. Comparison of Numerical and Simulation Results

We evaluated the analytical model by correlating it to the ns-3 simulator. In order to make this comparison, we used different RAW slot durations and different MCSes, as shown in Table 4. We also ran simulations enabling capture effect (CE), to compare results from the analytical model with a more realistic scenario where higher energy packets may be captured in case of a collision.

**Figure 4:**
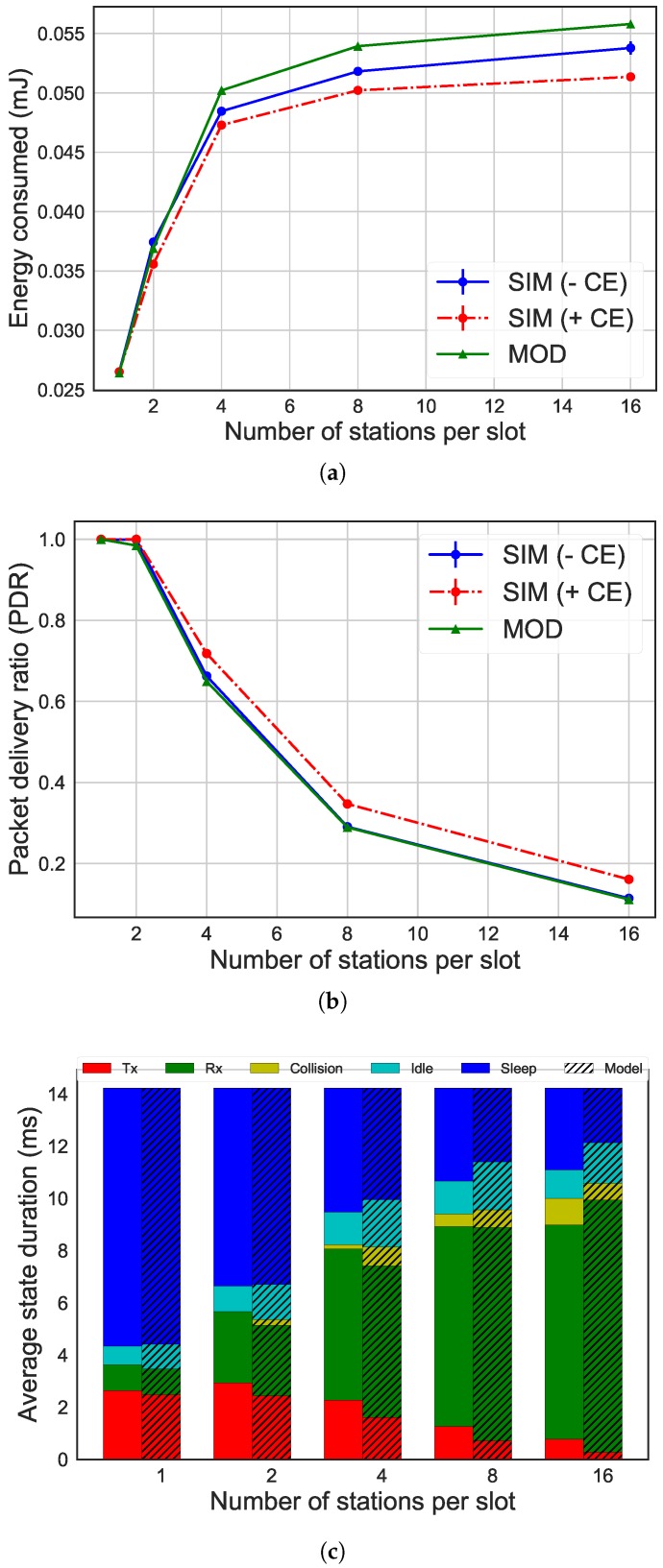
Comparison of results between simulation without capture effect (CE) (simulation (SIM) − CE) and with CE (SIM + CE) and analytical model (MOD) for 300 kbps, 16 bytes as payload size and 16,384 μs as slot duration. (**a**) average energy consumption per STA; (**b**) packet delivery ratio (PDR); (**c**) radio state duration per station (STA).

**Figure 5:**
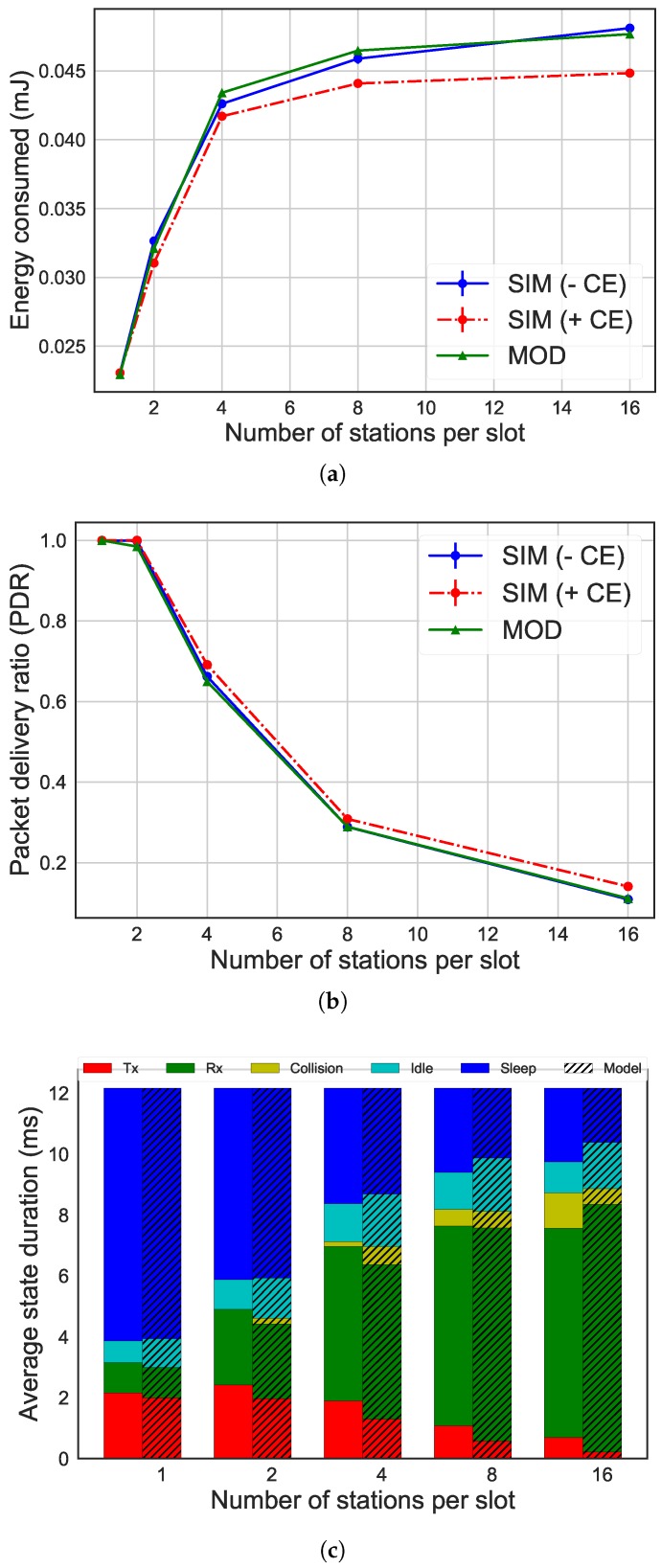
Comparison of results between simulation without CE (SIM − CE) and with CE (SIM + CE) and analytical MOD for 600 kbps, 64 bytes as payload size and 14336 μs as slot duration. (**a**) average energy consumption per STA; (**b**) PDR; (**c**) radio state duration per STA.

**Figure 6:**
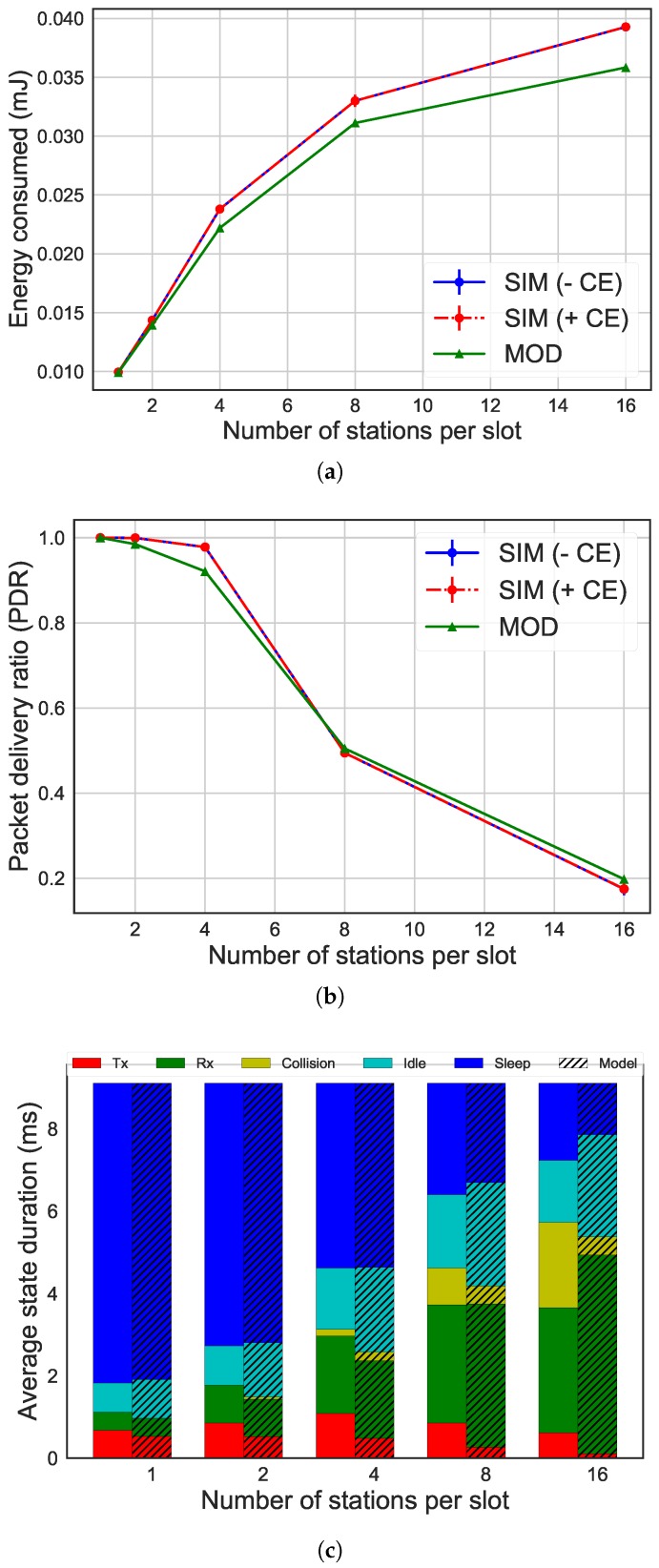
Comparison of results between simulation without CE (SIM − CE) and with CE (SIM + CE) and analytical MOD for 4 Mbps, 64 bytes as payload size and 11264 μs as slot duration. (**a**) average energy consumption per STA; (**b**) PDR; (**c**) radio state duration per STA.

Figure 4, Figure 5 and Figure 6 give an overview of the differences between the results of the ns-3 simulator and the analytical model, considering different MCSes. Specifically, the evaluation of our energy model has been done by comparing the average energy consumption, the number of packets successfully transmitted and the time spent in each radio state on average by the STAs. The graphs show how performance (y-axis) is influenced by the number of STAs per slot (x-axis). In the graphs regarding the average energy consumption and the packets successfully transmitted, there is the comparison between the results of the simulations with ns-3 without CE, the simulations with CE enabled, and the model. The graph about the radio state duration per slot shows the comparison between the results of the simulations without CE (column on the left) and of the Markov model (column on the right, with stripes). The left bar always shows the time spent in each of the five states in the simulation experiments, while the right bar shows the time spent in each state as calculated by the Markov model.

Even using different MCSes, the results of our analytical model were similar to simulation. Moreover, a high number of STAs reduced accuracy. The discrepancy started from 1% for the energy consumption in scenarios with two STAs per slot and 300 kbps, increasing to 4% with 16 STAs and 300 kbps and 9% for 4 Mbps. Regarding the number of packets transmitted, the difference went from 0% with two STAs for all the MCSes up to 14% with 16 STAs for 4 Mbps. The difference was caused by the use of a simplified model to represent the IEEE 802.11ah network. In fact, the analytical model did not consider the collision between more than two STAs, while the simulator did. This difference can be noted by the fact that the time the STA spends in the collision state is less in the analytical model results than in the ns-3 simulator results, being the cause of the difference in the energy consumption (as it can be seen in Figure 4c). As a result, the inaccuracy of the model was higher as the number of STAs increases due to higher collision probability. A similar effect occurs for higher data rates.

Also, other factors such as CE were not taken into account in the model. The comparison between the analytical model and simulations enabling CE shows a 7% difference of the energy consumption when considering 16 STAs and 600 kbps. This difference increased up to 9% for 16 STAs and 4 Mbps. The effect of CE decreased as the data rate increased, as more STAs were located at the same distance form the AP, reducing the chance of a significant difference in receive power.

A more complete overview of all the results for different payload sizes and slot durations can be seen in Table 5, Table 6 and Table 7. These tables show the results for different MCSes. For each MCS, we ran simulations with different slot and packet sizes. In the first two columns, there are the slot size and the payload sizes, while the remaining columns show the results over simulations with 16 STAs. From this overview, it can be seen how in general payload size does not affect accuracy as much as slot duration and MCS do.

In conclusion, in terms of energy consumption, on average our model shows at average a difference of the 5% when compared to simulations with and without CE, specifically the average difference without CE is 3%, while with CE it increases to the 6%.

**Figure 7:**
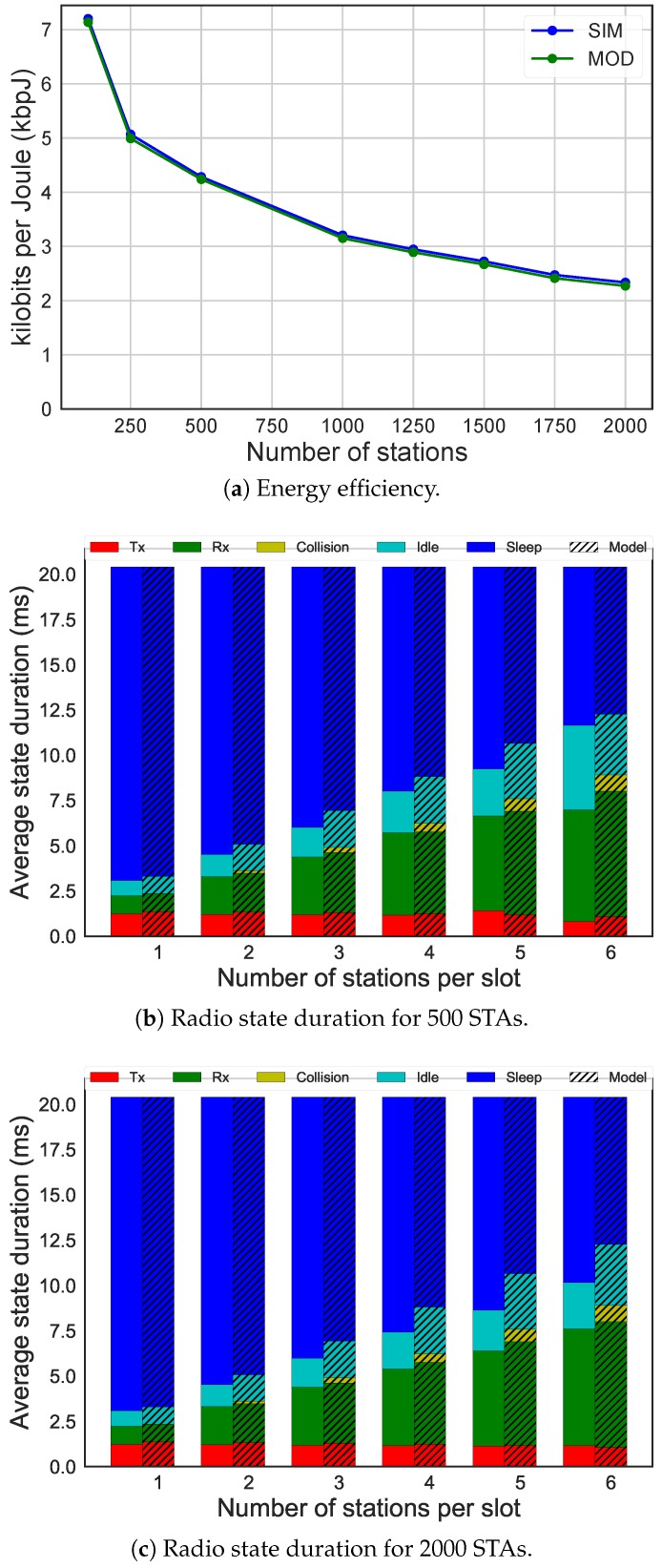
Comparison of results between SIM and analytical MOD for 600 kbps, 16 bytes payload size, 60 s packet arrival interval, 10 groups, and five slots per group.

The previous results compared performance of a single RAW slot. To show wider applicability of the analytical model, Figure 7 compares the analytical model to simulation, for a full system simulation over a 2000s period. It considers RAW configuration of 10 groups with 5 slots each. Both CE and CSB are disabled, and MCS1 at 1 MHz (data rate of 600 kbps) is considered. The beacon interval is set to 1024 ms and the Poisson distributed packet arrival interval to 60 s with 16 byte payload size. As the analytical model calculated the energy consumption and number of transmissions per slot, we calculated the energy consumption and number of successful transmissions over the entire evaluation period by calculating these values for slots with zero up to eight contending STAs, and multiplying it by the number of slots of each type that occurred. Moreover, we added the sleep energy of the stations outside of their assigned slots and the energy to receive the beacon. The energy to receive the beacon considerably affected the results, so even if there was a difference in the state duration, it was not noticeable in Figure 7a. The difference in performance stayed below 3%, which verified the results presented above for individual slots. It also showed the applicability of the model to full system evaluation.

### 5.3. Evaluation of Energy Efficiency of RAW

#### 5.3.1. Evaluation of Different Grouping Configurations

It has already been shown how the RAW mechanism has an impact on network performance and how bad configurations can negatively impact throughput, latency, and energy efficiency [22]. In this work, we focus on understanding which parameters influence the energy consumption in power constrained devices using RAW.

The following results show the behaviour of the energy efficiency and the latency considering different RAW grouping configurations, i.e., using a different number of slots per group, and traffic scenarios, as shown in Table 8. We consider scenarios from low traffic where the STAs send packets with an average packet arrival interval around 1500–3600 s, medium traffic where the STAs send packets with an average packet arrival interval around 600 s, to a high traffic having an average packet arrival interval around 1–30 s, with various grouping parameters, using ten groups and a different amount of slots, from one to five per group. We also compare to traditional CSMA/CA. In the following graphs, we provide the energy efficiency in terms of bits per Joule, representing the total number of payload bits that can be transmitted with 1 Joule of energy. A higher value represents better energy efficiency [29]. Latency is defined as the average time between a packet entering the transmit buffer of the STA and arriving at the network layer of the AP.

**Figure 8:**
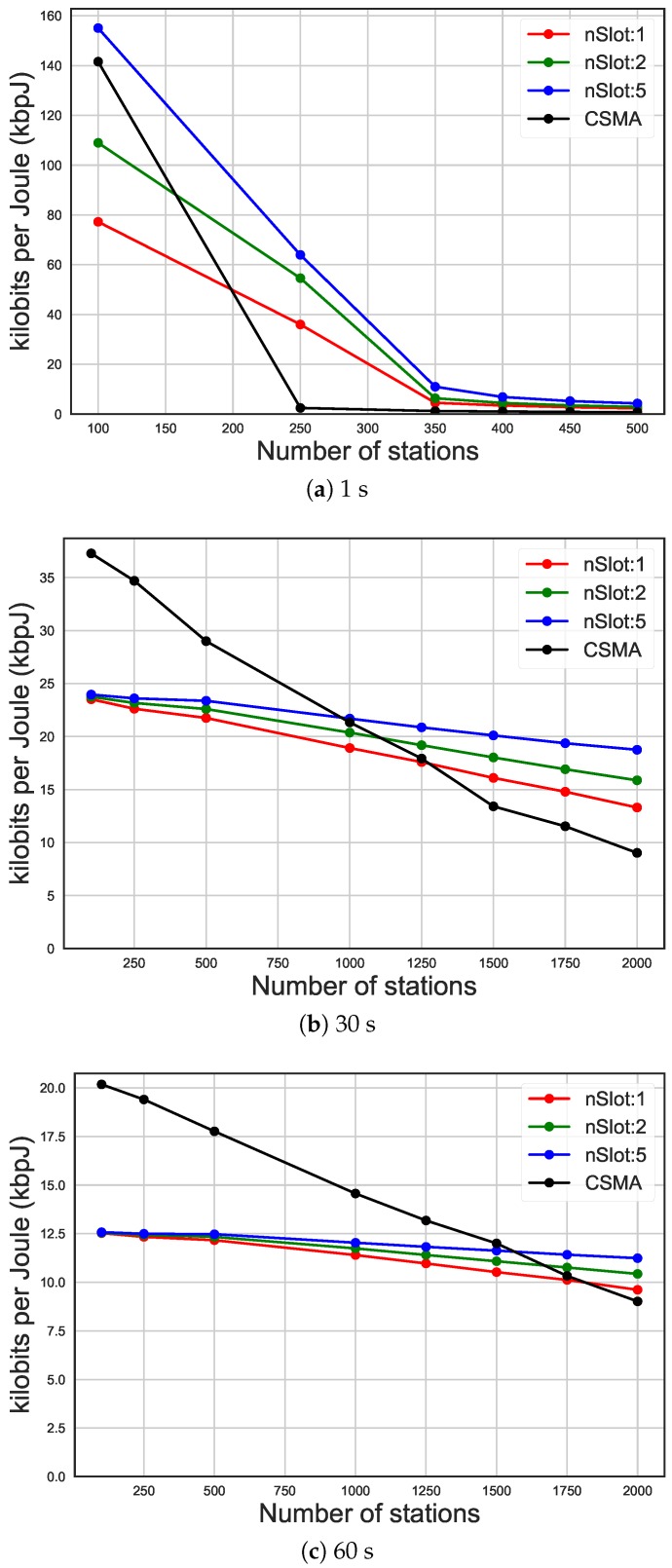
Energy efficiency of different packet arrival intervals, comparing carrier-sense multiple access with collision avoidance (CSMA/CA) and the usage of 10 RAW groups and one, two and five slots per group.

**Figure 9:**
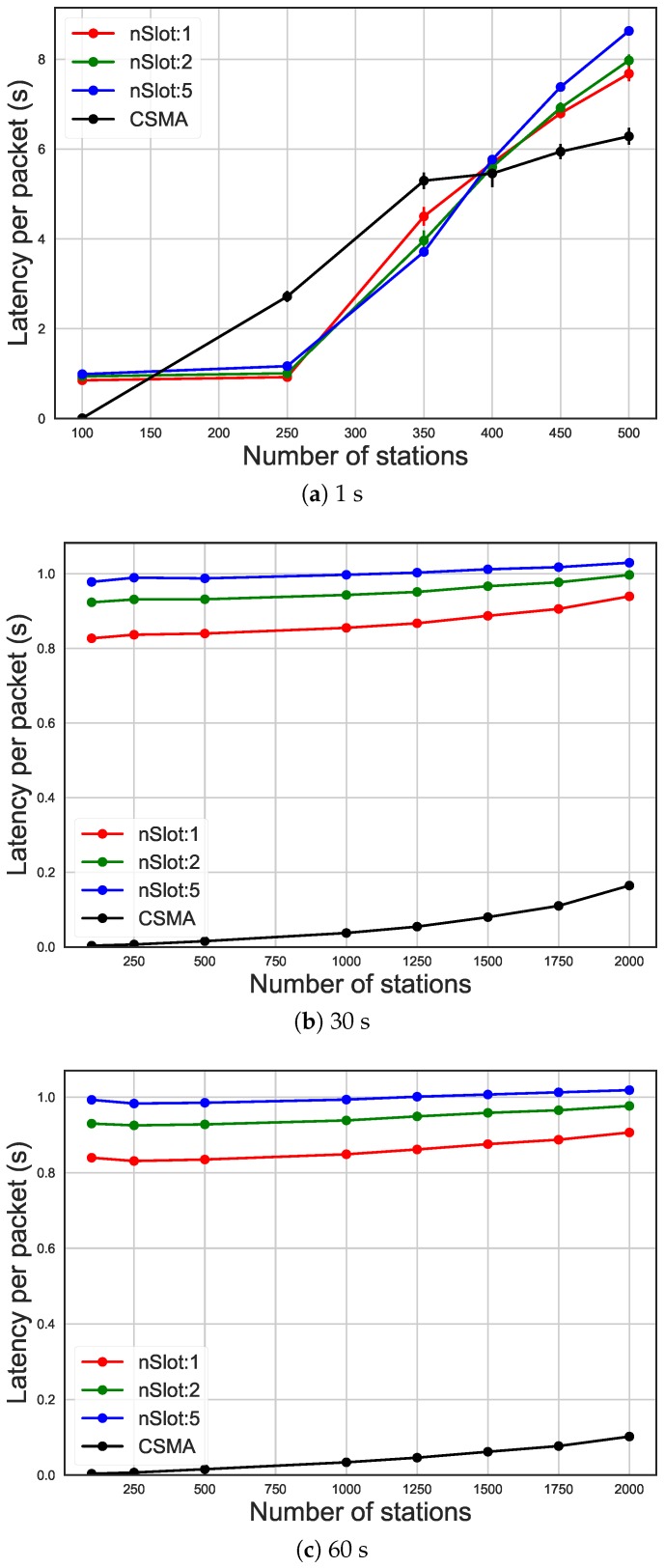
Latency of different packet arrival intervals, comparing CSMA/CA and the usage of 10 RAW groups and 1, 2 and five slot per group.

As it can be seen in Figure 8 and Figure 9, the trend of the average energy efficiency and the latency (on the y-axis) is a function of the number of STAs (x-axis) and it is influenced by the current traffic rate. In fact, it can be noted how the use of different numbers of RAW slots or the usage of CSMA/CA affects energy efficiency. In case of high traffic (average packet arrival interval of 30 s and more than 1250 STAs, in Figure 8b), increasing the total number of slots to 50 leads to 40% increase in energy efficiency compared to only ten slots and over 100% compared to CSMA/CA. In high traffic, as it can also be seen in Figure 8a, using a higher number of slots leads to better energy efficiency, even if due to saturation the performance drops. On the other hand, the number of slots does not affect energy efficiency when the traffic in the network is medium or low. In fact, as it can be seen in Figure 8b,c, when the number of STA and the traffic were low, the energy efficiency was poorly affected by the number of slots and CSMA/CA has better energy efficiency compared to RAW. However, having more slots led to higher latency, as can be seen in Figure 9b,c. In fact, each slot received an equal time-share of the beacon interval, so having more slots means they were smaller. This leads, when slots are bigger, to a higher probability for a packet to arrive during its STA’s assigned slot, and therefore to be sent during the same beacon interval. That is why having more slots leads to a higher latency, as STAs have to wait longer until they can access their slot. In case of high traffic (average packet arrival interval of 30s and more than 1250 STAs, in Figure 9b), increasing the total number of slots to 50 leads to 10% increase in latency compared to only ten slots and six times compared to CSMA/CA. In Figure 9a, on the other hand, it can be seen how RAW leads to saturation slower than CSMA/CA, which is saturated already at 250 STAs, while RAW starts being saturated at 350 STAs. As such, the latency of CSMA/CA is actually higher in this case.

**Figure 10:**
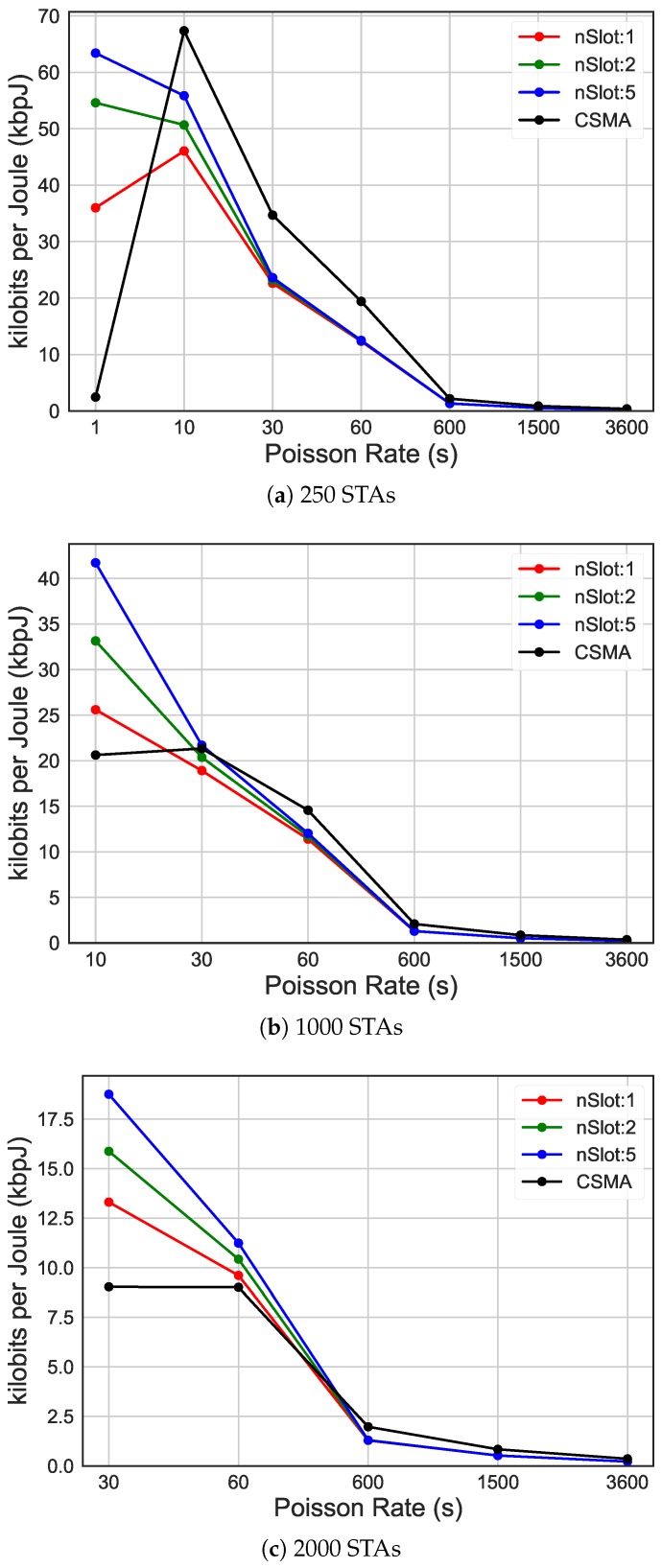
Energy efficiency of different traffic rates, comparing CSMA/CA and the usage of 10 RAW groups and 1, 2 and 5 slot per group.

**Figure 11:**
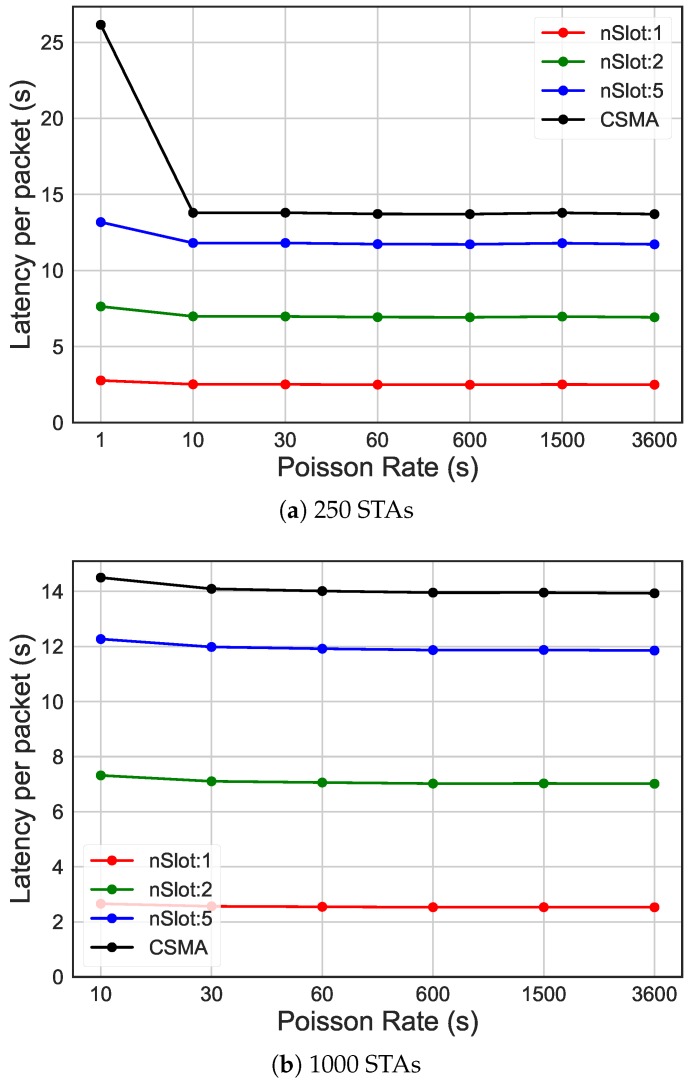
Latency of different traffic rates, comparing CSMA/CA and the usage of 10 RAW groups and one, two and five slots per group.

In Figure 10, these results show the impact of RAW compared to CSMA/CA on the energy efficiency (y-axis), considering networks with different traffic rates, expressed by average packet arrival intervals (x-axis). In case of high traffic, as it can be seen in Figure 10a with average packet arrival interval of 1s, increasing the number of slots to 50 leads to 76.15% increase in energy efficiency compared to only ten slots and over 2000% compared to CSMA/CA. When the traffic rate is higher than 600s, as it can be seen in Figure 10b,c, this difference is not so dramatic anymore, due to less contention. However, as it can be seen in Figure 11, different grouping configurations lead to a dramatic impact on latency, independently of the traffic load.

**Figure 12:**
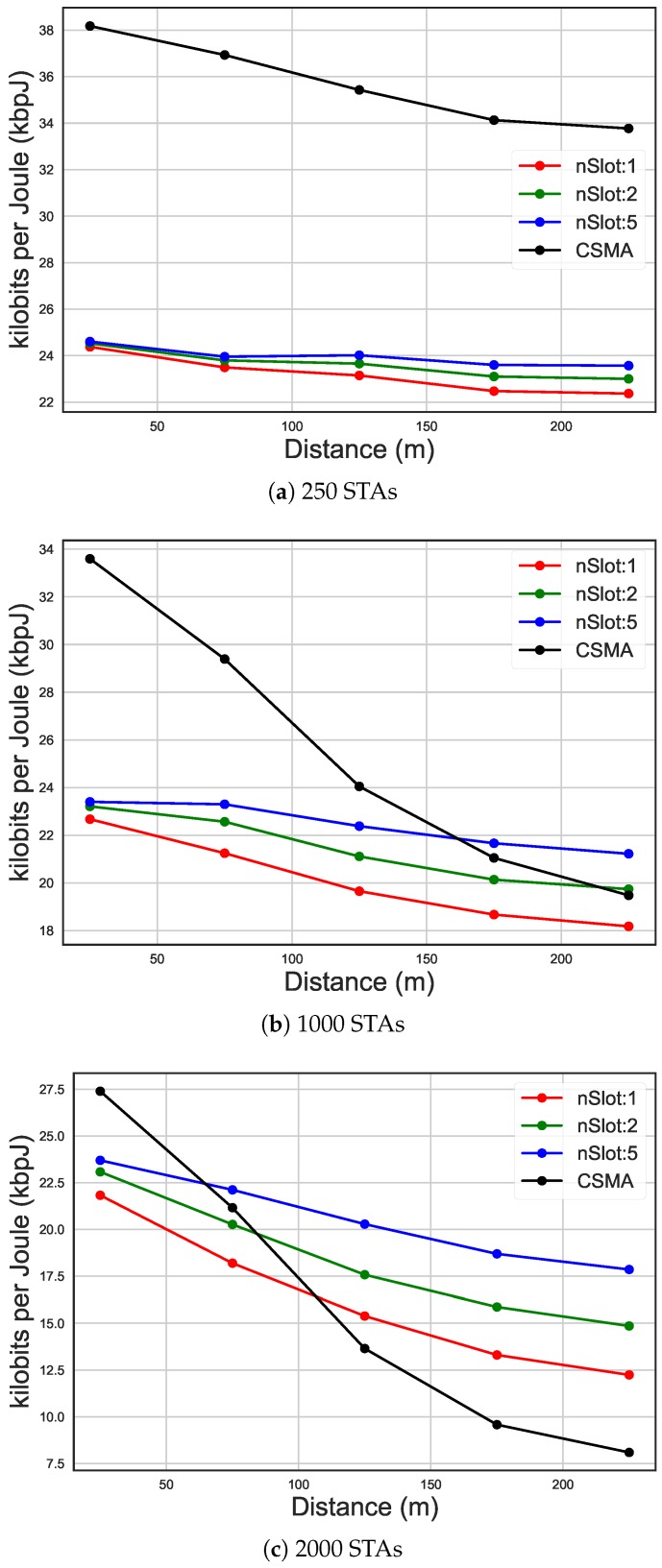
Energy efficiency on distance, comparing CSMA/CA and the usage of 10 RAW groups and one, two and five slots per group, using 30 s as average packet arrival interval.

**Figure 13:**
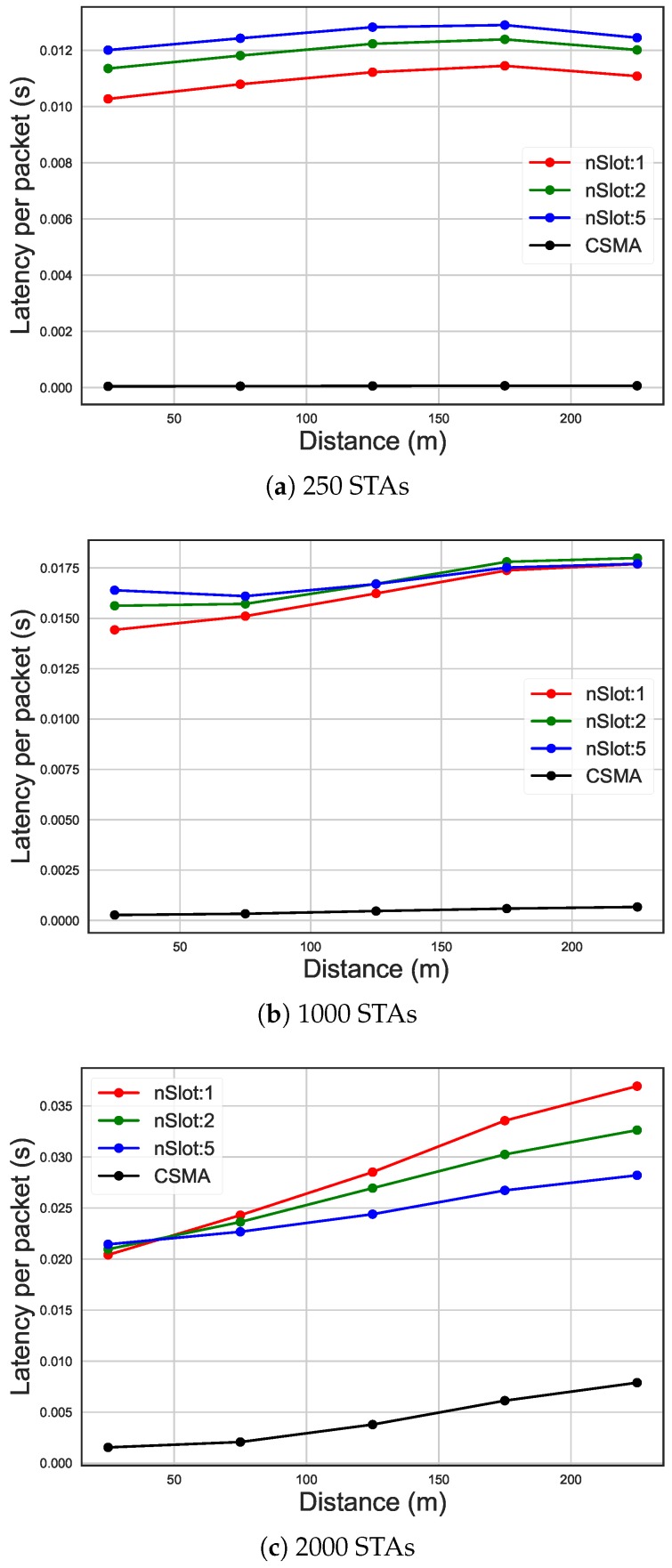
Latency on distance, comparing CSMA/CA and the usage of 10 RAW groups and one, two and five slots per group, using 30 s as average packet arrival interval.

Figure 12 shows the average energy consumption of STAs placed at various distances from the AP. In all these scenarios it can be seen how distance affects energy consumption, considering the same traffic ratio. Namely, energy efficiency decreased with distance. This was because, due to CE, STAs that are placed further away from the AP in case of collision are disadvantaged compared to the closer ones. In fact, in case of a collision, the AP chose the signal with more power. However, in the case of less STAs in the network, CSMA/CA outperformed RAW because the amount of traffic was low and so is the amount of contention. When contention is high, RAW clearly provides more fairness, decreasing the difference in energy efficiency between close and far away STAs compared to CSMA/CA. However, as can be seen in Figure 13, CSMA/CA has lower latency compared to RAW because of lower contention due to less traffic. However, as for energy consumption, in case of higher contention, latency grew faster than some RAW grouping configurations.

#### 5.3.2. Evaluation of Different Beacon Interval Durations

In this section, we compare the energy efficiency, calculated as bits per Joule, and the latency per packet for different beacon intervals. For these simulations, we used the configuration in Table 3. On the top of that, we have used different beacon intervals (204.8 ms, 409.6 ms, 1024 ms, 2048 ms), ten RAW groups, five slots per RAW group, 16 bytes as payload size. The packets arrive with an average arrival interval of 30 s.

**Figure 14:**
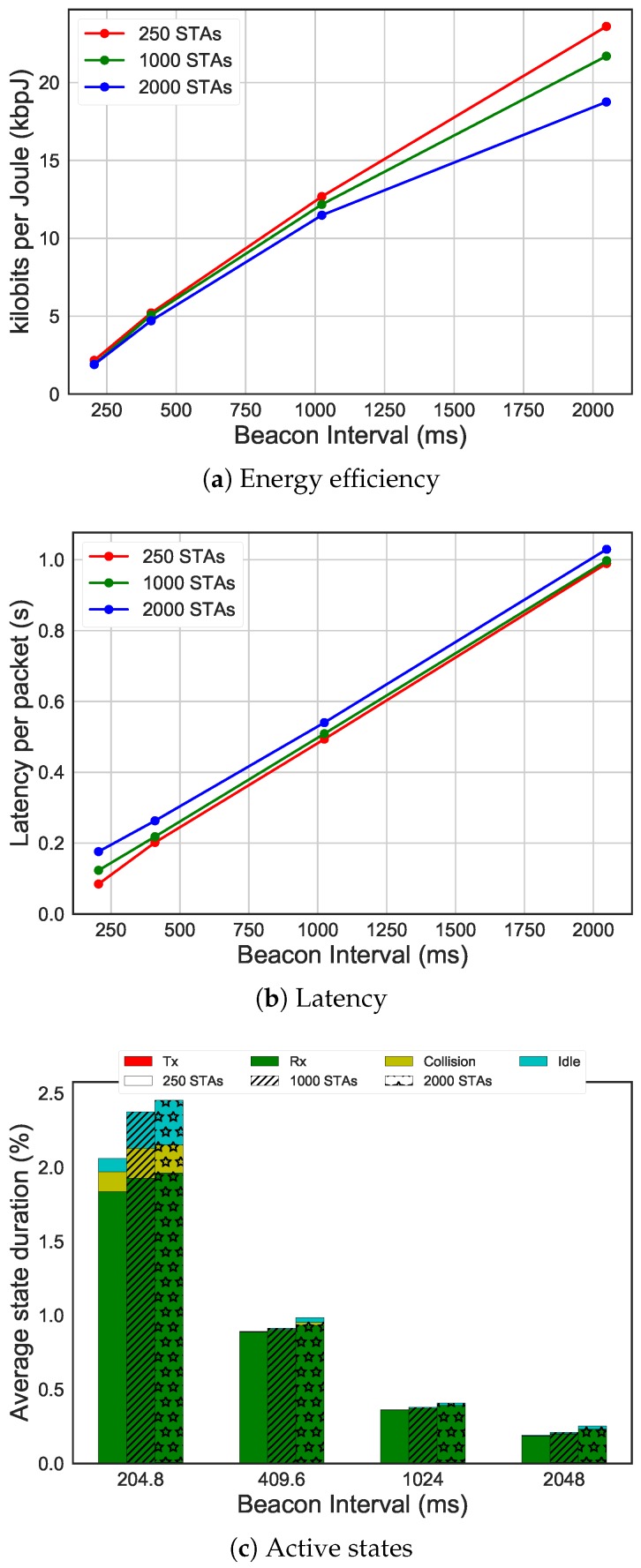
Energy efficiency and latency comparing different beacon intervals, using 16 bytes of payload size, average packet arrival interval of 30 s, 10 RAW groups and five slots per group.

The graph in Figure 14a shows the comparison of bit per Joule between different numbers of STAs having different beacon intervals (on the x-axis). The difference in performance for different numbers of STA was mainly due to collisions between the STAs sharing the same slots. In fact, simulations with less STAs had better energy efficiency than the ones with more STAs, as we have already shown before. However, the beacon interval duration linearly improves energy efficiency, but at the cost of increased latency, as shown in Figure 14b. The latency time was, on average, half of the beacon interval. STAs spent most of their time, around 98%, in the sleep state, however Figure 14c gives an overview of how much time STAs spend in active states (i.e., receiving, transmitting, colliding, idling). With the increase of beacon interval, since STAs sleep more, there is an increase of transmitting time in relation to the total active time. This is probably due to the bigger slot size.

#### 5.3.3. Evaluation of Different Packet Sizes

In this section, we compare the energy efficiency and latency as functions of packet size. For these simulations, we used the configuration in Table 3. On the top of that, we used different payload sizes (32, 64, 128, 256, 512 bytes), 10 RAW groups, five slots per RAW group, 2048ms as beacon interval. The packets arrive with an average arrival interval of 30s.

**Figure 15:**
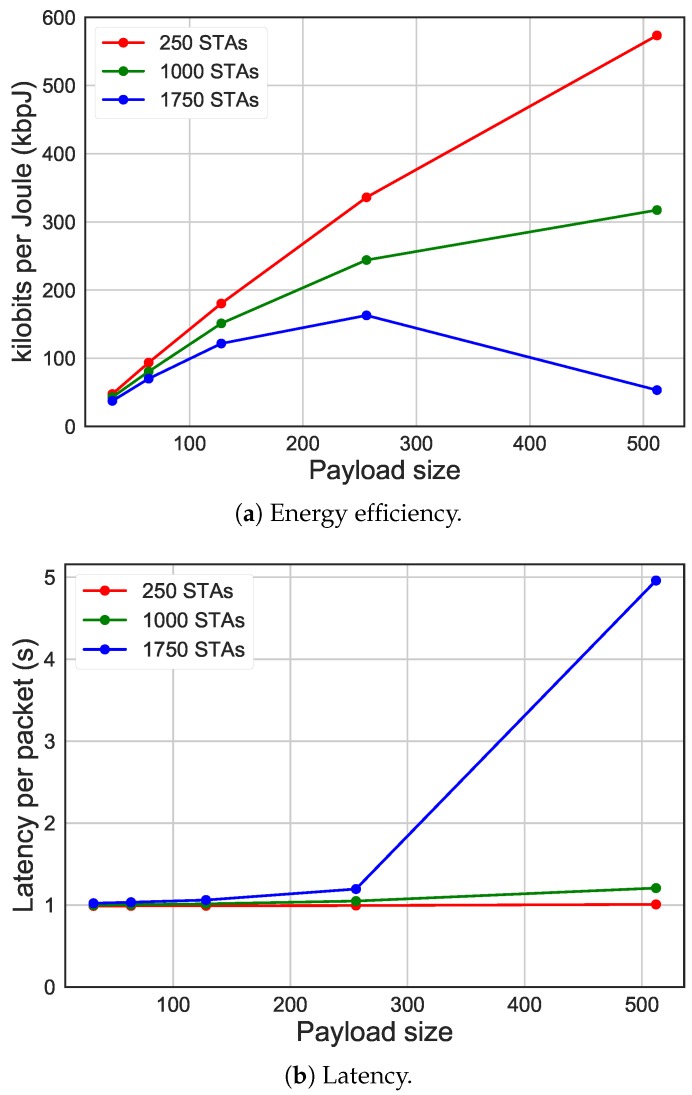

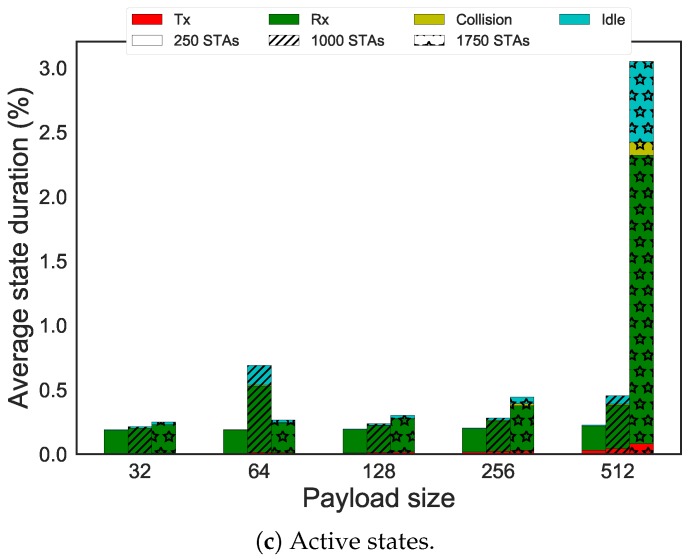
Energy efficiency and latency comparing different payload sizes, using 2048 ms beacon interval, average packet arrival interval of 30 s, 10 RAW groups and five slots per group.

The graph in Figure 15a shows the comparison in terms of bit per Joule (y-axis) between different number of STAs having different payload sizes (on the x-axis). These results show that there was a linear improvement in terms of bit per Joule with the increase of payload size, until saturation occurs. However, bigger payload sizes lead faster to network saturation, when more STAs are in the network, due to the higher amount of time STAs spend sending packets. As can be seen in Figure 15b, when having 1750 STAs and a payload size bigger than 256 bytes, the network starts being saturated, while with less STAs and less traffic, the latency did not increase dramatically with higher payload sizes. STAs spend most of their time in the sleep state, however Figure 15c gives an overview of how much time STAs spend in active states (i.e., receiving, transmitting, colliding, idling). With the increase of payload size, larger number of STAs lead to more collisions and lower auspicious transmission time due to saturation, in fact, simulations with a lower cost of STAs show a more linear increase of transmission time.

### 5.4. Energy Efficiency Using TWT

TWT is a functionality that allows STAs with low traffic to decrease their energy consumption by negotiating with the AP when the STAs will be awake in order to transmit their packets. This allows them to ignore intermediary beacons and save significant amounts of energy if they transmit sporadically. However, problems as the clock drift phenomenon can arise [30], which we leave out of consideration in this study.

In our simulator, we implemented TWT in order for the STAs to sleep and wake up at certain intervals, in this case corresponding to the transmission interval (i.e., when using TWT, the STAs wakes up at the same interval as the transmission time). We then evaluated the battery consumption, comparing it with the usage of only RAW. For the calculation of the battery-life we consider linear battery power consumption and we do not take into account battery aging and degradation. We have used the configuration in Table 3 and, on the top of that, the configuration in Table 9. To create a realistic scenario, we used transmission intervals starting from 30 s to 1 h. With these simulations we want to see the performance of the STAs not having to wake up to receive all the beacons, assessing the impact of beacon overhead.

**Figure 16:**
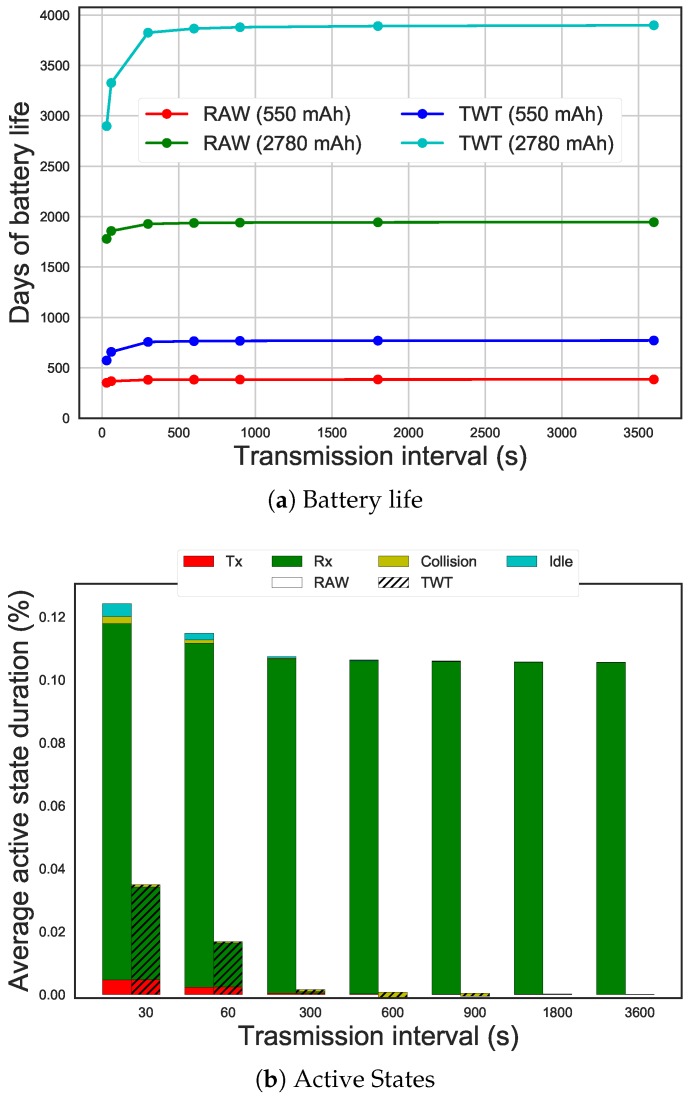
Battery-life using RAW and TWT with different transmission intervals.

Figure 16a shows the comparison of days of battery-life between the use of RAW and TWT, with 100 STAs sending packets at different transmission intervals (on the x-axis). When STAs transmit every hour, the battery-life, considering coin cell batteries of 550 mAh of charge, can reach 770 days, while for RAW it is around 385 days. This is a twofold improvement. TWT can reach around 3897 days considering AA batteries of 2780 mAh, while RAW reaches 1945 days. Figure 16b shows the amount of time STAs spend in active states on average (i.e., receiving, transmitting, colliding, idling). It can be seen how STAs using RAW spend most of their time in the receiving state and STAs using TWT do not. This is because STAs using TWT are allowed to skip beacons, resulting in lower energy consumption.

In TWT there is a 35% increase in battery life when increasing the transmission interval from 30 s to 1 h. But with RAW, the difference is only 10%, as energy consumption is mostly dominated by beacon reception. However, for transmission intervals between 5 and 60 min the improvement is limited to 1%, due to the dominant energy consumption of the sleep state.

## 6. Conclusions

We presented a fourfold contribution. First, we extended the analytical model of IEEE 802.11ah RAW in order to provide the calculation of energy consumption.

Second, we have evaluated our model in comparison to a realistic network simulator. The comparison showed that our model is accurate when considering networks where STAs send one packet per RAW slot, no hidden nodes, and no CE, with a maximum deviation of 7% from the simulation in terms of energy efficiency.

Third, we provided an evaluation of the energy consumption of the RAW mechanism of IEEE 802.11ah using different configurations and traffic scenarios, showing how different grouping configurations affect energy efficiency depending on traffic rates, on distance and on the number of STAs. This has been done by the implementation of an energy model for IEEE 802.11ah in the ns-3 simulator. We have shown how having more RAW slots always results in better energy efficiency, however, when having low traffic in the network CSMA/CA outperforms RAW. Specifically, we have seen this behavior when the average packet arrival interval was higher than 30s and the number of STAs was higher than 1250. However, using more slots leads to a higher latency, so in applications where the latency is critical, it is advisable to use fewer slots or pure CSMA/CA. When energy consumption is more important, the use of RAW leads to higher energy efficiency.

Finally, we extended the ns-3 simulator adding the TWT feature, and we compared it to the usage of the RAW mechanism, showing how the sleeping time dominates the battery-life when having longer transmission intervals and how the reception of beacons affects the energy consumption significantly. In fact, we have shown how the usage of TWT can increase the battery-life by 100% compared to RAW.

In conclusion, we have shown that RAW is a mechanism that is easily adaptable to many scenarios, especially with high traffic, where latency is not critical. Optimal RAW operation requires calculations mostly from the AP side. When the transmission time is known, TWT shall be used, since it improves the energy consumption by more than 100% compared to RAW if the transmission interval is 5 min or more.

## Figures and Tables

**Table 1:** Variables introduced in the Markov process model.

Variable Name	Description
$T_{slot}$	Total duration of the RAW slot
$T_{s}$	Duration of a successful transmission time slot
$T_{c}$	Duration of a collision time slot
$T_{tx}$	Duration of a frame transmission time slot
$T_{ack}$	Duration of an ACK reception time slot
$T_{e}$	Duration of an empty (i.e., back-off) time slot
$P_{idle}$	Power consumed when the radio is idle
$P_{tx}$	Power consumed when the radio is transmitting
$P_{rx}$	Power consumed when the radio is receiving
$P_{sleep}$	Power consumed when the radio is in the sleep state
*N*	Total number of STA in the RAW slot
$R_{max}$	STA re-transmission limit before discarding frame
$S_{p}$	Payload size
δ	MCS data rate
SIFS	Short Interframe Space (160 μs)
AIFS	Arbitrary Interframe Space (240 μs)

**Table 2:** The duration of the different radio states based on the IEEE 802.11ah standard.

Radio State	Duration
$T_{tx}$	$S_{p}÷\delta +AIFS$
$T_{ack}$	1000 μs
$T_{s}$	$T_{tx}+SIFS+T_{ack}$
$T_{c}$	$T_{s}-T_{ack}$
$T_{e}$	52 μs

**Table 3:** Default PHY and MAC layer parameters used in our experiments.

Parameter	Value
Transmission power	0 dBm
Transmission gain	0 dB
Reception gain	3 dB
Noise Figure	3 dB
Voltage	3.3 V
Propagation loss model	Outdoor, macro [28]
Error Rate Model	YansErrorRate
Wi-Fi mode	MCS1, 1 MHz
Maximal Distance from AP	250 m
Station distribution	uniformly random
Capture Effect	enabled
CWmin	15
CWmax	1023
MAC header type	legacy header
Queue size	10 packets
**Power Consumption**	**Value**
Receiving ($P_{rx}$)	92 mW
Idle ($P_{idle}$)	20 mW
Transmission ($P_{tx}$)	204 mW
Sleeping ($P_{sleep}$)	99 nW

**Table 4:** Configuration for the comparison of numerical and simulation results.

Parameter	Value
Number of RAW groups	1
Number of slots per group	1
Station distribution	circular
Distance from AP	1 m
Queue size	1 packet
Capture effect (CE)	disabled
Cross slot boundary (CSB)	disabled
**Low Data Rate (300 kbps)**	**Value**
Wi-Fi mode	MCS0, 1 MHz
MAC Payload size	16 bytes, 64 bytes
RAW slot duration	16,384 μs, 32,768 μs (16 bytes);
	21,504 μs, 43,008 μs (64 bytes)
**Medium Data Rate (600 kbps)**	**Value**
Wi-Fi mode	MCS1, 1 MHz
MAC Payload size	16 bytes, 64 bytes
RAW slot duration	12,288 μs, 23,552 μs (16 bytes);
	14,336 μs, 28,672 μs (64 bytes)
**High Data Rate (4 Mbps)**	**Value**
Wi-Fi mode	MCS9, 1 MHz
MAC Payload size	16 bytes, 64 bytes, 256 bytes
RAW slot duration	11,264 μs, 21,504 μs (16 bytes);
	11,264 μs, 21,504 μs (64 bytes);
	12,288 μs, 24,576 μs (256 bytes)

**Table 5:** Overview of the comparison between analytical model, simulation and simulation with capture effect (CE) for 300 kbps, 16 stations (STAs) per slot.

		Model		Simulation without CE		Simulation with CE	
**Slot Duration (μs)**	**Payload**	**PDR**	**E (mJ)**	**PDR**	**E (mJ)**	**PDR**	**E (mJ)**
16,384	16 bytes	0.11	0.06	0.11	0.05	0.16	0.05
32,768	16 bytes	0.22	0.12	0.27	0.12	0.39	0.11
21,504	64 bytes	0.11	0.08	0.11	0.07	0.16	0.07
43,008	64 bytes	0.21	0.16	0.27	0.16	0.39	0.15

**Table 6:** Overview for 600 kbps, 16 STAs per slot.

		Model		Simulation without CE		Simulation with CE	
**Slot Duration (μs)**	**Payload**	**PDR**	**E (mJ)**	**PDR**	**E (mJ)**	**PDR**	**E (mJ)**
12,288	16 bytes	0.11	0.04	0.12	0.04	0.15	0.04
23,552	16 bytes	0.22	0.09	0.25	0.09	0.32	0.08
14,336	64 bytes	0.11	0.05	0.11	0.05	0.14	0.04
28,672	64 bytes	0.22	0.10	0.25	0.11	0.32	0.10

**Table 7:** Overview for 4 Mbps, 16 STAs per slot.

		Model		Simulation without CE		Simulation with CE	
**Slot Duration (μs)**	**Payload Size**	**PDR**	**E (mJ)**	**PDR**	**E (mJ)**	**PDR**	**E (mJ)**
11,264	16 bytes	0.21	0.04	0.18	0.04	0.18	0.04
21,504	16 bytes	0.24	0.07	0.39	0.08	0.39	0.08
11,264	64 bytes	0.20	0.04	0.17	0.04	0.18	0.04
21,504	64 bytes	0.24	0.07	0.36	0.08	0.36	0.08
12,288	256 bytes	0.17	0.04	0.16	0.04	0.16	0.04
24,576	256 bytes	0.24	0.09	0.35	0.09	0.35	0.09

**Table 8:** Configuration for the evaluation of restricted access window (RAW) energy consumption.

Parameter	Value
Beacon Interval	2048 ms
Payload size	16 bytes
Average packet interval arrival	1 s, 10 s, 30 s, 60 s, 600 s, 3600 s
Number of RAW groups	10
Number of slots per group	1, 2, 5
Cross slot boundary (CSB)	enabled

**Table 9:** Configuration for the comparison of energy consumption using target wake time (TWT).

Parameter	Value
Beacon Interval	2048 ms
Average packet arrival interval	30 s, 1 m, 5 m, 10 m, 15 m, 30 m, 1 h
Number of RAW groups	1
Number of slots per group	1
Battery capacity	550 mAh, 2780 mAh

## References

[1] Micheletti M., Mostarda L., Piermarteri A. Rotating Energy Efficient Clustering for Heterogeneous Devices (REECHD). Proceedings of the 2018 IEEE 32nd International Conference on Advanced Information Networking and Applications (AINA).

[2] Tian L., Deronne S., Latré S., Famaey J. Implementation and Validation of an IEEE 802.11Ah Module for Ns-3. Proceedings of the Workshop on Ns-3.

[3] Tian L., Sljivo A., Santi S., De Poorter E., Hoebeke J., Famaey J. Extension of the IEEE 802.11ah ns-3 Simulation Module. Proceedings of the Workshop on ns-3.

[4] Khorov E., Krotov A., Lyakhov A. Modelling machine type communication in IEEE 802.11ah networks. Proceedings of the IEEE International Conference on Communication Workshop (ICCW).

[5] Zhou Y., Wang H., Zheng S., Lei Z.Z. Advances in IEEE 802.11ah standardization for machine-type communications in sub-1GHz WLAN. Proceedings of the IEEE International Conference on Communications Workshops (ICC).

[6] Sun W., Choi M., Choi S. (2013). IEEE 802.11ah: A long range 802.11 WLAN at sub-1GHz. J. ICT Stand..

[7] Adame T., Bel A., Bellalta B., Barcelo J., Oliver M. (2014). IEEE 802.11ah: The WiFi approach for M2M communications. IEEE Wirel. Commun..

[8] Khorov E., Lyakhov A., Krotov A., Guschin A. (2015). A survey on IEEE 802.11ah: An enabling networking technology for smart cities. Comput. Commun..

[9] Park M. (2015). IEEE 802.11ah: Sub-1-GHz license-exempt operation for the internet of things. IEEE Commun. Mag..

[10] Baños-Gonzalez V., Afaqui M.S., Lopez-Aguilera E., Garcia-Villegas E. (2016). IEEE 802.11ah: A technology to face the IoT challenge. Sensors.

[11] Raeesi O., Pirskanen J., Hazmi A., Levanen T., Valkama M. Performance evaluation of IEEE 802.11ah and its restricted access window mechanism. Proceedings of the IEEE International Conference on Communications Workshops (ICC).

[12] Beltramelli L., Österberg P., Jennehag U., Gidlund M. Hybrid MAC Mechanism for Energy Efficient Communication in IEEE 802.11ah. Proceedings of the IEEE International Conference on Industrial Technology (ICIT).

[13] Nawaz N., Hafeez M., Zaidi S.A.R., McLernon D.C., Ghogho M. Throughput enhancement of restricted access window for uniform grouping scheme in IEEE 802.11ah. Proceedings of the IEEE International Conference on Communications (ICC).

[14] Qutab-ud din M., Hazmi A., Badihi B., Larmo A., Torsner J., Valkama M. Performance analysis of IoT-enabling IEEE 802.11ah technology and its RAW mechanism with non-cross slot boundary holding schemes. Proceedings of the IEEE 16th International Symposium on A World of Wireless, Mobile and Multimedia Networks (WoWMoM).

[15] Tian L., Khorov E., Latré S., Famaey J. (2017). Real-Time Station Grouping under Dynamic Traffic for IEEE 802.11ah. Sensors.

[16] Park M. IEEE 802.11ah: Energy efficient MAC protocols for long range wireless LAN. Proceedings of the IEEE International Conference on Communications (ICC).

[17] Zhao Y., Yilmaz O.N.C., Larmo A. Optimizing M2M Energy Efficiency in IEEE 802.11ah. Proceedings of the IEEE Globecom Workshops (GC Wkshps).

[18] Zheng L., Ni M., Cai L., Pan J., Ghosh C., Doppler K. (2014). Performance Analysis of Group-Synchronized DCF for Dense IEEE 802.11 Networks. IEEE Trans. Wirel. Commun..

[19] Khorov E., Lyakhov A., Yusupov R. Two-Slot Based Model of the IEEE 802.11ah Restricted Access Window with Enabled Transmissions Crossing Slot Boundaries. Proceedings of the IEEE 19th International Symposium on A World of Wireless, Mobile and Multimedia Networks (WoWMoM).

[20] Sljivo A., Kerkhove D., Tian L., Famaey J., Munteanu A., Moerman I., Hoebeke J., De Poorter E. (2018). Performance Evaluation of IEEE 802.11ah Networks With High-Throughput Bidirectional Traffic. Sensors.

[21] Bel A., Adame T., Bellalta B. (2018). An energy consumption model for IEEE 802.11ah WLANs. Ad Hoc Netw..

[22] Tian L., Famaey J., Latré S. Evaluation of the IEEE 802.11ah Restricted Access Window mechanism for dense IoT networks. Proceedings of the IEEE 17th International Symposium on A World of Wireless, Mobile and Multimedia Networks (WoWMoM).

[23] Bellalta B. (2016). IEEE 802.11ax: High-efficiency WLANS. IEEE Wirel. Commun..

[24] Afaqui M.S., Garcia-Villegas E., Lopez-Aguilera E. (2017). IEEE 802.11ax: Challenges and Requirements for Future High Efficiency WiFi. IEEE Wirel. Commun..

[25] Khorov E., Kiryanov A., Lyakhov A., Bianchi G. (2018). A Tutorial on IEEE 802.11ax High Efficiency WLANs. IEEE Commun. Surv. Tutor..

[26] (2017). IEEE Standard for Information technology–Telecommunications and information exchange between systems—Local and metropolitan area networks–Specific requirements—Part 11: Wireless LAN Medium Access Control (MAC) and Physical Layer (PHY) Specifications Amendment 2: Sub-1GHz License Exempt Operation. IEEE Std 802.11ah-2016 (Amendment to IEEE Std 802.11-2016, as Amended by IEEE Std 802.11ai-2016).

[27] Atmel Corporation Atmel AT86RF215 Device Family. http://ww1.microchip.com/downloads/en/devicedoc/atmel-42415-wireless-at86rf215_datasheet.pdf.

[28] Bellekens B., Tian L., Boer P., Weyn M., Famaey J. Outdoor IEEE 802.11ah Range Characterization Using Validated Propagation Models. Proceedings of the IEEE Global Communications Conference (GLOBECOM).

[29] Rodoplu V., Meng T.H. (2007). Bits-per-Joule Capacity of Energy-Limited Wireless Networks. IEEE Trans. Wirel. Commun..

[30] Bankov D., Khorov E., Lyakhov A., Stepanova E. Clock Drift Impact on Target Wake Time in IEEE 802.11ax/ah Networks. Proceedings of the International Conference Engineering & Telecommunication (En&T).

